# The use of race and ethnicity in air pollution epidemiology and methodological recommendations: A scoping review focusing on California

**DOI:** 10.1097/EE9.0000000000000485

**Published:** 2026-06-01

**Authors:** Arnab K. Dey, Chen Chen, Vivian Do, Shirley (Kexin) Zhou, Raul Gonzalez, Tarik Benmarhnia

**Affiliations:** a Scripps Institution of Oceanography, University of California San Diego, La Jolla, California; b Department of Population Health Sciences, University of Utah, Salt Lake City, Utah; c Huntsman Cancer Institute, University of Utah, Salt Lake City, Utah; d Department of Earth, Ocean, and Atmospheric Science, Faculty of Science, University of British Columbia, Vancouver, British Columbia, Canada; e Irset Institut de Recherche en Santé, Environnement et Travail, UMR-S 1085, Inserm, University of Rennes, EHESP, Rennes, France

**Keywords:** Air pollution, Race and ethnicity, Methodology, Mediation analysis, Effect modification, Exposure disparity, Confounding

## Abstract

Air pollution remains a significant global health risk, and exposure to air pollutants can lead to multiple health issues across the life course. Air pollution is also an environmental justice issue and plays a crucial role in health disparities across racial and ethnic groups, through both differential exposure and differential vulnerability. Historical patterns of discriminatory siting of emission sources have led to differential exposure to air pollution among racial and ethnic groups. Differential vulnerability, influenced by social factors and community composition, further exacerbates health inequities. Given these complex mechanisms through which race and ethnicity (RE) influence both exposure and vulnerability to air pollution, it is important to understand how RE is operationalized in air pollution epidemiology and identify methodological opportunities. We conducted a scoping review to summarize the use of RE in air pollution epidemiology literature with a focus on California. From January 2000 to July 2025, we identified a total of 178 publications, excluding those that only used RE as a confounder. The number of studies exploring air pollution disparity across RE or RE as effect modifiers for the air pollution-outcome relationship increased over time, but the number of publications exploring the mediating role of air pollution in outcome disparity across RE remained low. We identified several methodological practices that warrant further consideration and provided corresponding methodological recommendations that can help researchers and policymakers better understand current practices for consideration of RE in air pollution-related health studies.

**What this study adds**

This study enhances our understanding of existing methodological approaches to using race and ethnicity in air pollution epidemiology. By systematically reviewing how race and ethnicity are operationalized and utilized in California-focused studies, we identified practices that may lead to biased conclusions about environmental health inequities. Our findings reveal that while studies examining pollution disparities across racial groups have increased, research exploring air pollution’s mediating role in health disparities remains limited. We also provided methodological recommendations for researchers and policymakers to better understand and address environmental justice issues, ultimately supporting effective interventions to reduce health inequities across diverse communities.

## Introduction

In 2019, air pollution was the fourth largest risk factor for premature death globally, accounting for 6.7 million premature deaths.^1^ Exposure to air pollution has been linked to increases in risk for all-cause, cardiovascular, and respiratory mortality and morbidity by many epidemiological studies.^2–8^ Several studies have also highlighted disparities in exposure and vulnerability to air pollution across race and ethnicity (RE).^9–11^ Given the different definitions of vulnerability across disciplines, we explicitly define vulnerability in this manuscript as the increased health risk of an individual at a given exposure level, originating from socioeconomic, environmental, and personal factors, and excluding the impact of differential exposure to hazards. While public and government agencies in the United States have increasingly focused on environmental justice issues,^12–14^ research on the health effects of air pollution has been criticized for lacking appropriate consideration of racial and ethnic inequities.^15,16^

Historically, health inequities across racial and ethnic groups have been recognized and recorded in the United States since the founding of colonial America.^17^ In this context, race is recognized not as a biological construct reflecting innate differences, but as a social construct that captures the social classification of people and the subsequent impacts of racism in a race-conscious society. This classification has a profound impact on daily life experiences through interpersonal interactions and serves as a proxy for the historical and contemporary structural factors that perpetuate inequities.^18^ The role of environmental determinants, like air pollution, in such health inequities received more attention in the past decades when the environmental justice movement emerged following the Warren County landfill protest in 1982, when civil rights activists organized against the dumping of polychlorinated biphenyls in the predominantly Black community in North Carolina.^11^ Since then, many studies, focused specifically on air pollution, documented disparities in both the exposure^11,19,20^ and vulnerability^21,22^ to air pollution across racial and ethnic groups.

Differential exposure results from discriminatory siting of emission sources, likely fueled by lower costs for building industrial facilities near minority communities, weaker opposition due to limited social capital of such communities, discriminatory zoning from the early 1900s, and practices of systemic and codified mortgage discrimination and segregation (e.g., redlining), which have contributed to residential patterns and concentrated marginalized groups in environmentally burdened areas.^11^ On the other hand, differential vulnerability, where air pollutants affect individuals and communities differently based on racial and ethnic factors, often results from historical and structural racism that manifests through poor built environments, high personal exposure, and internal dose from the same ambient pollution due to activity patterns, inadequate healthcare access, and intense psychosocial stress.^17,21,23^ Previous studies have demonstrated that disregarding either differential exposure or differential vulnerability will lead to an underestimation of the health disparity.^24^

**Figure 1.**
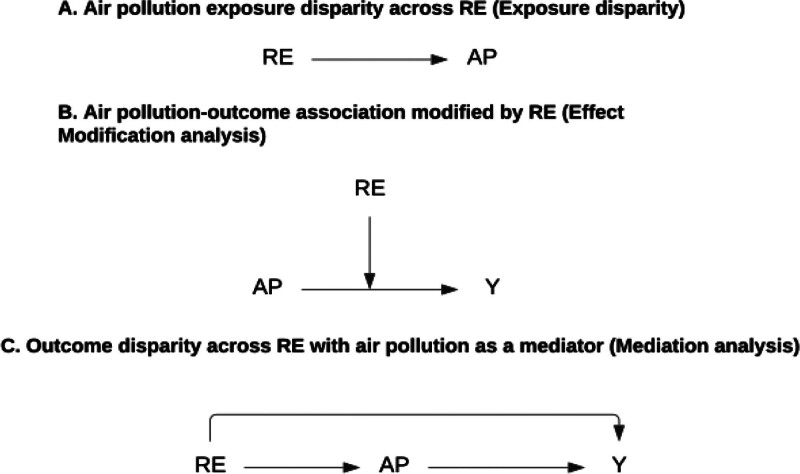
Schematic figure depicting the study types for air pollution studies considering race and ethnicity. Schematic depiction C could also be interpreted as treating RE as the confounder of AP and Y relationship, which is not a study type of interest in this study due to its preclusion of systematic racism manifested through AP. AP indicates air pollution; RE, race and ethnicity; Y, can be any outcome

Given these complex mechanisms through which RE influences both exposure and vulnerability to air pollution, it is important to understand how RE is operationalized in air pollution epidemiology.^15,25^ Benmarhnia et al^26^ discuss the inferential and methodological challenges and provide a framework to systematically study the use of RE in these contexts. Briefly, in air pollution-related health studies, RE has been used as confounder, effect modifier, or main exposure of interest^26^ (Figure 1). Considering RE as a confounder is the most common usage of RE in air pollution-related health studies^27^ and acknowledges that differential exposure and health disparities exist across racial and ethnic groups. However, treating RE as a confounder regards potential differential vulnerability leading to such disparities (e.g., systematic racism manifested through air pollution) as irrelevant to the research question through a “ritualistic adjustment,”^28–30^ and precludes consideration of differential vulnerability across RE groups. However, such consideration can help identify the most vulnerable subpopulations and shape the appropriate interventions.

When RE is considered an effect modifier (Figure 1B) in the association between air pollution and a health outcome (including stratified analysis by RE or interaction terms between air pollution and RE), both differential exposure and differential vulnerability are incorporated into the analysis, but the challenge remains in the interpretation of root causes for the observed disparity in the health effect of air pollution across RE. A recent review of 10 US-based empirical air pollution-related health studies that considered RE from 2016 to 2022 found a lack of in-depth discussion of such disparity.^10^ Perry et al connected this lack of conceptual discussion to the origin of mainstream environmental health studies, in which RE was usually considered as a biological determinant and the observed health disparity was considered evidence of inferiority of the racial and ethnic minorities and not a proxy of addressable factors such as structural racism.^16^

When RE is used as the main exposure of interest, the study could either focus on quantifying the differential exposure (i.e., including air pollution as the outcome without considering health) (Figure 1A) or on decomposing the total effect of RE on a health outcome to an indirect part mediated through differential exposure to air pollution and a direct part representing the effect of RE through other pathways (Figure 1C). Proper interpretation of the observed disparity in air pollution is a challenge for the first type of study where the focus is on quantifying differential exposure, and incorporation of a conceptual framework is crucial. Studies focusing on mediation/decomposition analysis ask a simple question: how would disparities in the health outcome change if disparities in the mediator (e.g., air pollution) were removed?^31^ Although causal decomposition analysis is gaining popularity in mainstream epidemiological studies as it combines intervention and health disparity considerations through the counterfactual framework, its usage remains rare in air pollution-related health studies.^25^ Besides, there are many assumptions and challenges in the application of decomposition analysis that impedes its application, including incorporation of clear causal framework and the interpretation around the manipulability of RE.^26^

Given these methodological challenges, we aimed to summarize how RE are operationalized in air pollution epidemiology studies and provide methodological recommendations. We conducted a scoping review of air pollution-related health studies in California, focusing on their use of RE. California offers an ideal case study as the most populated US state with significant air quality challenges—it has the highest number of counties exceeding the Environmental Protection Agency’s air quality standards^32,33^ for ambient fine particulate matter (PM) and ozone. It also experiences an increasing wildfire smoke pollution that contributes significantly to total air pollution concentrations and the PM composition in the state.^34,35^ With its diverse population (40% Latino, 35% white, 15% Asian/Pacific Islander, 5% Black)^36^ and progressive air pollution control policies,^37,38^ California has been central for studies that advance understanding of the role of RE in health disparity studies. Understanding the operationalization of RE in California air pollution-related health studies could provide relevant insights for all US regions. We provided researchers with methodological recommendations and help policymakers, and the public better understand current practices for the consideration of RE in air pollution-related health studies.

## Methods

Based on the PRISMA Extension for Scoping Review,^39^ we conducted the scoping review in five steps: eligibility criteria establishment, initial search for eligible articles, abstract and title screening, full-text review, and data extraction. At least two researchers participated in each step and consensus was achieved in every step.

We focused on three major types of studies (Figure 1): air pollution exposure disparity study across RE (exposure disparity analysis), air pollution-outcome association modified by RE (effect modification [EM] analysis), and outcome disparity across RE with air pollution as a mediator (mediation analysis). We did not include articles that only considered RE as a confounder for the air pollution-health outcome relationship due to the high prevalence of such practice and the preclusion of evaluating systematic racism manifested through air pollution.^30^

We created a list of eligibility criteria and a list of exclusion criteria to guide the process (Table S1; https://links.lww.com/EE/A433). To restrict the review to a manageable size, we decided a priori to focus on ambient air pollutants, including the six criteria air pollutants,^40^ wildfire-related air pollutants, and air quality index (AQI). We did not impose any restriction on the health outcomes and included studies that aimed to evaluate the differential exposure to air pollutants across RE. We also limited our review to articles published between 1 January 2000 to 31 July 2025 and conducted specifically within California or in the United States but with locations within California (e.g., studying and evaluating US cities, some of which are in California).

**Figure 2.**
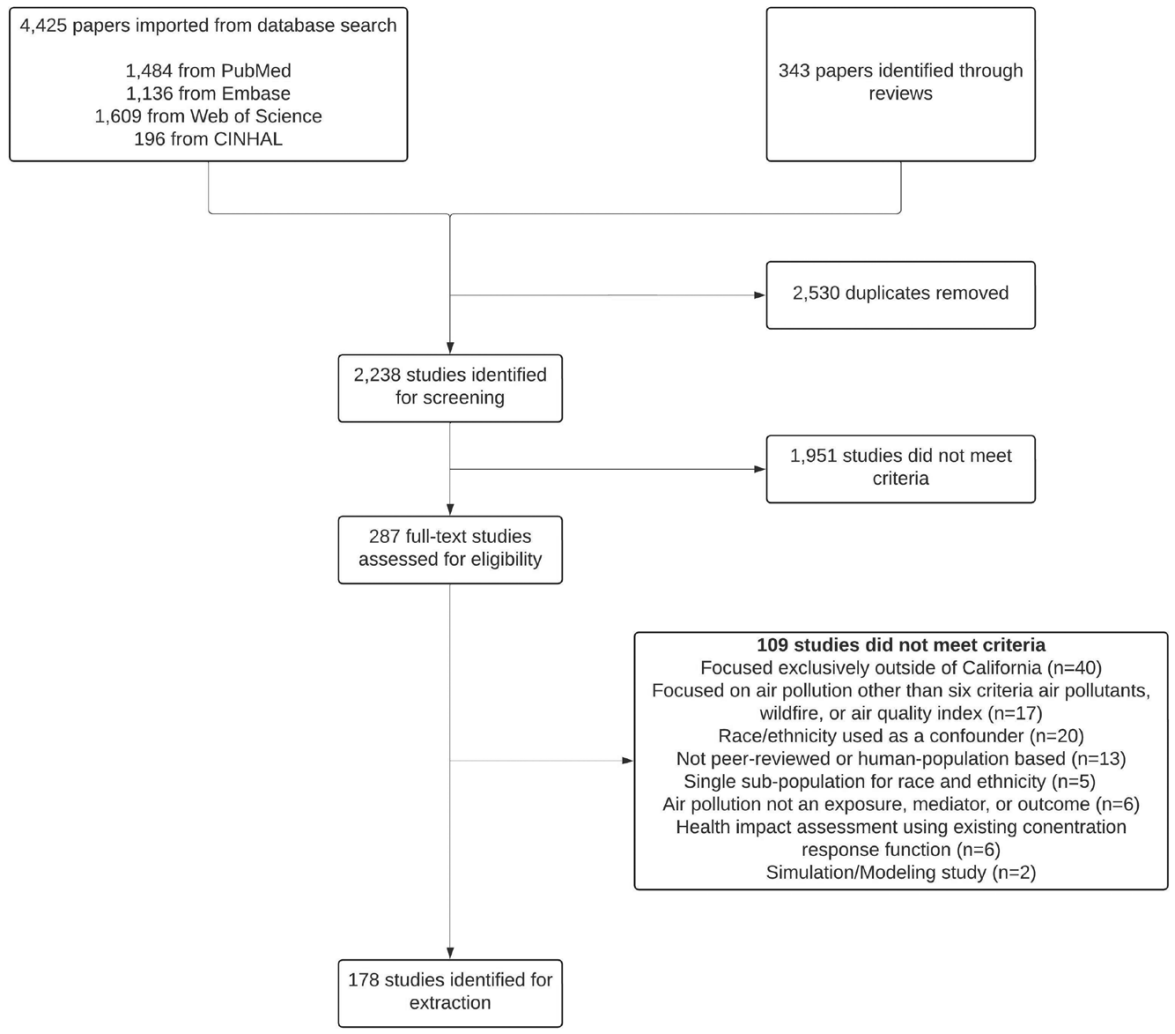
Flowchart of the scoping review and articles excluded at each stage.

Based on the eligibility criteria, we conducted searches in four databases (PubMed, Embase, Web of Science, and CINHAL). Briefly, we created separate sets of search terms for air pollution, RE, geographical areas, and article type (Table S2; https://links.lww.com/EE/A433). We then applied these search terms to search for the title and abstract, and identified articles satisfying all sets of terms simultaneously, with manual restrictions on the publication dates. To increase the comprehensiveness of our initial search, we also added potentially eligible original articles cited in relevant reviews and California Air Resources Board reports to our initial search results, with the relevant reviews identified in a similar manner as the main search (Table S3 and Supplementary text 1; https://links.lww.com/EE/A433). This supplemental process identified 343 original articles (Figure 2), the large majority of which were already captured by the primary database searches and removed as duplicates.

Before beginning the formal abstract and title screening process, two screeners completed a thorough training exercise using 10 research articles on similar topics that were not included in the study sample. Articles selected for inclusion by both screeners were sent for full-text review, while articles deemed ineligible by both screeners were excluded. When either screener voted “maybe” or their votes differed, the article was marked as a conflict and was reviewed by a separate conflict resolver to decide whether the article should be excluded or sent for full-text review (Figure S1; https://links.lww.com/EE/A433). At the full-text review stage, two researchers independently read and voted for the included articles, with conflicts resolved through discussion.

We created a data extraction form to collect information on basic study characteristics and important methodological considerations for the final articles.^25,26,41^ We extracted detailed methodological considerations including the study design, the specific role of RE in the analysis (as an exposure, mediator, or effect modifier), the spatial and temporal resolution of air pollution exposure, the statistical techniques used to evaluate disparities, and whether the authors utilized a conceptual framework to interpret findings related to structural racism or environmental injustice. The questions included in the extraction are included in Supplementary material (Supplementary text 3; https://links.lww.com/EE/A433). Two researchers independently extracted information from each article and reached consensus on discordant items via discussion. For studies conducting mediation analysis, we assessed three major methodological concerns based on the guideline for reporting mediation analyses (AGReMA statement), which was not integrated in the extraction form.^42^

## Results

The initial search strategy yielded 4425 nonmutually exclusive articles from the four databases (1484 from PubMed, 1136 from Embase, 1609 from Web of Science, and 196 from CINHAL) (Figure 2). We also added 343 original articles identified through relevant reviews and California Air Resources Board reports. Among these 4768 articles, we excluded 2530 duplicates and included 2238 articles for abstract and title screening, 287 for full-text review, and 178 for data extraction. Details of exclusion at the full-text review stage are included in Supplementary Text 2; https://links.lww.com/EE/A433.

### Basic study characteristics

Among 178 studies extracted, 105 studies only conducted EM analysis (i.e., considered RE as an effect modifier; in the association between air pollution and a health outcome; Figure 1B), and 64 studies only examined exposure disparity (i.e., utilized RE as the primary predictor to quantify differential air pollution burdens across groups, without incorporating health outcomes; Figure 1A). Of the remaining nine studies, six only conducted mediation analysis (i.e., decomposed the total effect of RE on a health outcome to an indirect part mediated through differential exposure to air pollution and a direct part representing the effect of RE through other pathways; Figure 1C),^43–48^ one examined both exposure disparity and mediation,^49^ and two conducted both EM and mediation analyses.^50,51^

Studies with consideration of RE other than confounder increased over the years, from 9 (5.1%) published between 2000 and 2010, 53 (29.8%) published between 2011 and 2020, and 116 (65.2%) published within the short span of January 2021–July 2025. The growth was particularly noticeable for exposure disparity studies (n = 65), which increased from two studies in 2000–2010 to 46 studies in 2021–2025. Studies assessing EM showed a similar trend (n = 107), increasing from seven studies in 2000–2010 to 67 studies in 2021–2025, while mediation analyses emerged primarily after 2011 (Table 1).

**Table 1.** Number of studies published by type of study and periods

Period of publication	Exposure disparity studies (N = 65)	EM analysis studies (N = 107)	Mediation analysis studies (N = 9)
2000–2010	2^a^ (3.1%)	7 (6.5%)	1^a^ (11.1%)
2011–2020	17 (26.2%)	33 (30.8%)	3 (33.3%)
2021–2025	46 (70.8%)	67^b^ (62.6%)	5^b^ (55.6%)

a One study conducting both exposure disparity analysis and mediation analysis.

b Two studies conducting both EM and mediation analysis.

#### Use of RE variables across study types

RE data mostly came from individual medical records (66 studies, 37.1%), followed by area-level aggregates from general population surveys such as the American Community Survey (56 studies, 31.5%), and individual reports from cohorts (35 studies, 19.7%) (Table 2). Self-reported RE was the predominant collection method (104 studies, 58.4%), though notably, 69 studies (38.8%) did not discuss their collection method.

**Table 2.** Sources and characteristics of race and ethnicity data across study types

Study characteristics	All studies (N = 178^a^)	Exposure disparity studies (N = 65)	EM analysis studies (N = 107)	Mediation analysis studies (N = 9)
Sources of RE data				
Medical record (e.g., cancer registry, hospitalization)	66 (37.1%)	1 (1.5%)	63 (58.9%)	3 (33.3%)
General population survey (e.g., American Community Survey, Decennial Census Survey)	56 (31.5%)	45 (69.2%)	9 (8.4%)	2 (22.2%)
Data collected within the cohort (e.g., enrollment questionnaire)	35 (19.7%)	7 (10.8%)	26 (24.3%)	4 (44.4%)
Data product (e.g., Health Place Index, CalEnviroScreen)	16 (9.0%)	11 (16.9%)	5 (4.7%)	0 (0.0%)
Not discussed	5 (2.8%)	1 (1.5%)	4 (3.7%)	0 (0.0%)
Method of collection RE data				
Self-report	104 (58.4%)	57 (87.7%)	41 (38.3%)	8 (88.9%)
Not discussed	69 (38.8%)	7 (10.8%)	62 (57.9%)	1 (11.1%)
Other^b^	5 (2.8%)	1 (1.5%)	4 (3.7%)	0 (0.0%)
Type of RE data				
Individual level	108 (60.7%)	9 (13.8%)	95 (88.8%)	7 (77.8%)
Area level				
Proportion of RE within area/population	65 (36.5%)	53 (81.5%)	12 (8.4%)	0 (0.0%)
Metrics of structural racism	3 (1.7%)	1 (1.5%)	0 (0.0%)	2 (22.2%)
Composite index	2 (1.1%)	2 (3.1%)	0 (0.0%)	0 (0.0%)
Combined RE categories for analysis				
Yes	84 (47.2%)	33 (50.8%)	49 (45.8%)	4 (50.0%)
No	14 (7.9%)	8 (12.3%)	6 (5.6%)	1 (12.5%)
Unclear	80 (44.9%)	24 (36.9%)	52 (48.6%)	4 (44.4%)

a Three studies were classified into two study types simultaneously, leading to differences in total number of studies and sum of studies across study types in some rows.

b The other category represents a combination of administrative and patient self-reports, assessment by coroner or reported by a relative of the deceased, and response by a caregiver.

Regarding the specific type of RE data utilized, 108 studies (60.7%) used individual-level RE data and 65 studies (36.5%) used proportions of different RE groups within an area or population. Three studies utilized metrics aiming to capture dimensions of structural racism, such as Theil’s H index for racial segregation and the index of concentration at the extremes for racial polarization. Two studies used area-level composite scores that incorporated multiple aspects of demographic and socioeconomic characteristics (e.g., Social Vulnerability Index and CalEnvironGreen). The type of RE data used in studies also differed significantly by study type. Exposure disparity studies primarily used area-level proportions (53 of 65 studies, 81.5%), while EM studies predominantly used individual-level data (95 of 107 studies, 88.8%). (Table 2). Regarding RE categories utilized in the study, 84 studies (47.2%) explicitly reported collapsing specific groups or dropping certain categories from the analysis altogether, while for 80 studies (44.9%), the processing of RE categories was unclear. This practice varied widely: some studies collapsed all minoritized groups into a single “non-White” or “People of Color” category, while others combined specific subgroups, such as merging Asian and Native Hawaiian/Pacific Islander individuals or grouping various Hispanic/Latino identities into a single category. Furthermore, several studies dropped specific categories—most frequently American Indian, Alaska Native, or “Other” groups—from analysis due to insufficient sample sizes for statistical inference (Table 2).

Among studies that used either individual or proportion of RE groups in an area (n = 173, excluding the five studies that used composite index or metrics of segregation), “Asian, Black, Hispanic, White” and “Hispanic, non-Hispanic Black, non-Hispanic White” were the most commonly used categories, with each set of categorizations appearing in 10 (5.8%) studies. These were followed by “Black, White, Other” and “Hispanic, non-Hispanic Asian, non-Hispanic Black, non-Hispanic White” classifications that appeared in 7 (4.0%) studies each. Other common combinations were “Black, White” and “Hispanic, non-Hispanic Black, non-Hispanic White, Other” that were used in 6 (3.5%) studies. Five studies used “Black and non-Black” and the same number of studies used “non-Hispanic Black and non-Hispanic White” classifications. Some studies employed more specific categorizations like “Black, Cuban, Dominican, Hispanic, Hispanic Black, Hispanic White, Mexican, Puerto Rican, White,” while others used binary classifications such as “People of Color, White” or “non-minority, RE minority” (Table S4; https://links.lww.com/EE/A433).

To capture the multilevel nature of RE usage, we summarized whether additional RE variables were utilized in the study beyond the primary RE variable. We identified 38 studies (21.3%) that did so, with 25 studies providing justification for considering such additional variables. These justifications included using community-level RE proportions as confounders or region-level covariates in the second stage of a hierarchical model, accounting for residential segregation, or investigating spatial patterns to explain heterogeneity in associations. Others cited the need to address macro-social forces influencing neighborhood pollution over time.

#### Use of air pollution across study types

The most common approach to assess air pollution was statistical modeling (e.g., spatiotemporal statistical models) (70 studies, 39.3%), followed by dynamic model-based approaches (e.g., GEOS-Chem or CMAQ) and monitoring data alone (e.g., Air Quality System by the Environmental Protection Agency) (32 studies each, 18.0%), a combination of statistical and dynamic models fused through machine learning algorithms (23 studies, 12.9%), satellite data alone (e.g., TROPOMI or NOAA’s Hazard Mapping System) (four studies, 2.2%), and emission inventory data alone (two studies, 1.1%) (Table 3). Multiple methods were used in nine studies (5.1%), and the approach was not discussed in four studies (2.2%).

**Table 3.** Modeling methods and geographic units of the air pollutants included in the review by study type.

Study characteristics	All studies (N = 178^a^)	Exposure disparity studies (N = 65)	EM analysis studies (N = 107)	Mediation analysis studies (N = 9)
Sources of air pollution data				
Statistical modeling	70 (39.3%)	27 (41.5%)	40 (37.4%)	3 (33.3%)
Dynamic model-based approach	32 (18.0%)	12 (18.5%)	20 (18.7%)	1 (11.1%)
Monitoring data alone	34 (19.1%)	9 (13.8%)	24 (22.4%)	2 (22.2%)
Combination of statistical and dynamic model	23 (12.9%)	10 (15.4%)	12 (11.2%)	1 (11.1%)
Satellite data only	4 (2.2%)	3 (4.6%)	0 (0.0%)	1 (11.1%)
Emissions inventory data only	2 (1.1%)	2 (3.1%)	0 (0.0%)	0 (0.0%)
Multiple combinations^b^	9 (5.1%)	2 (3.1%)	7 (6.5%)	0 (0.0%)
Not discussed	4 (2.2%)	0 (0.0%)	4 (3.7%)	1 (11.1%)
Geographical units of air pollution data				
Administrative unit level				
Zip code/ZCTA	44 (24.7%)	3 (4.6%)	40 (37.4%)	1 (11.1%)
Census tract	32 (18.0%)	21 (32.3%)	9 (8.4%)	3 (33.3%)
County	22 (12.4%)	3 (4.6%)	18 (16.8%)	2 (22.2%)
Census block group	12 (6.7%)	10 (15.4%)	2 (1.9%)	0 (0.0%)
Census block	6 (3.4%)	5 (7.7%)	1 (0.9%)	0 (0.0%)
Air Basin	1 (0.6%)	0 (0.0%)	1 (0.9%)	0 (0.0%)
Other administrative units	11 (6.2%)	5 (7.7%)	6 (5.6%)	0 (0.0%)
Individual or residential address level	33 (18.5%)	2 (3.1%)	29 (27.1%)	3 (33.3%)
Grid cell level	11 (6.2%)	11 (16.9%)	0 (0.0%)	0 (0.0%)
Site-specific level	6 (3.4%)	5 (7.7%)	1 (0.9%)	0 (0.0%)

a Three studies were classified into two study types simultaneously, leading to differences in the total number of studies and the sum of studies across study types in some rows.

b This category represents studies that used multiple combinations of exposure sources and methods simultaneously, when multiple air pollutants were analyzed in one study or when comparison of multiple exposure modeling methods was conducted.

The geographical resolution of air pollution data includes administrative unit level (128 studies, 71.9%), individual or residential address level (33 studies, 18.5%), grid cell levels (11 studies, 6.2%), and site-specific level (six studies, 3.4%). Studies focusing on administrative levels mostly relied on ZIP Codes/ZCTAs (44 studies, 24.7%), census tracts (32 studies, 18.0%), and counties (22 studies, 12.4%) (Table 3). Spatial resolutions for studies using grid-based units varied substantially, ranging from 100 m to 48 km. Resolutions of air pollution assessment were typically driven by the underlying data sources or exposure models, such as the InMAP source-receptor matrix or HyADS.

Most studies examined a single pollutant (97 studies, 54.5%), while fewer studies investigated multiple pollutants: 38 studies (21.3%) examined two pollutants, 21 studies (11.8%) looked at three pollutants, and 22 studies (12.4%) analyzed four or more pollutants. 60%, 52.3%, and 44.4% of studies focused on single pollutant in exposure disparity analysis, EM, and mediation analysis, respectively (Table S5; https://links.lww.com/EE/A433). Studies utilizing the AQI or focusing specifically on wildfire smoke were categorized as single-pollutant analyses unless additional individual pollutants were also evaluated.

Across all 178 studies, PM was the most analyzed air pollutant, appearing in 135 studies (75.8%), with PM_2.5_ being the primary focus and analyzed in 130 studies. This was followed by NO_2_ (34.3%) and ozone (21.3%). Studies also assessed PM_2.5_ constituents (9.0%), wildfire-specific pollution (9.0%), AQI (1.1%), and other air pollutants such as NO_x_ and Benzene (10.7%). While PM dominated the research landscape, these specialized metrics provided additional context for source-specific and multipollutant exposures. To note, about half of the studies explored multiple air pollutants simultaneously, leading to higher than 100% when the studies were summed across air pollutants. In studies exploring exposure disparity (n = 65), PM_2.5_ and NO_2_ remained the most analyzed pollutants, utilized in 41 (63.1%) and 25 (38.5%) studies, respectively. For research involving EM analysis (n = 107), 83 (77.6%) included PM_2.5_, while 34 (31.8%) included NO_2_ (Table S6; https://links.lww.com/EE/A433).

### Methodological details by study type

#### Exposure disparity studies

Analytical approaches utilized in exposure disparity studies varied a lot. Among the studies that explored air pollution exposure disparities across RE (n = 65), descriptive metrics of air pollution (39 studies, 60.0%) and multivariable regression analyses (26 studies, 40.0%) were the most common analytical methods. Other analytical approaches included descriptive metrics of RE in areas with extreme air quality conditions (e.g., census tracts ranked in the highest 10 percentile of air pollution), univariable linear regression, hierarchical models, univariable quantile regression, clustering analysis, and visualization methods. Some studies utilized multiple analytical approaches, often combining descriptive statistics with more complex regression analyses to examine RE disparities in air pollution exposure.

#### Effect modification analysis studies

Among the studies (n = 107) that conducted EM, cohort (36 studies, 33.6%) and cross-sectional (26 studies, 24.3%) study designs were most common, followed by time-series (25 studies, 23.4%) and ecological (16 studies, 15.0%) study designs. Most studies (84 studies, 78.5%) explicitly mentioned EM or effect heterogeneity in their methods or results, while some only referred to statistical terms like interaction (4 studies, 3.7%) or stratification (19 studies, 17.8%). Most studies (70 studies, 65.4%) conducted formal heterogeneity tests to assess differences in effect estimates across RE groups with 47 studies observing significant heterogeneity. Of these studies, most (42 studies) reported stratum-specific results. However, among the 23 studies that did not detect heterogeneity, 20 studies still reported stratum-specific results.

Common analytical approaches utilized in EM studies included multivariable regression models (88 studies, 82.2%), hierarchical models (13 studies, 12.1%), and G-methods (nine studies, 8.4%). A few EM studies (five studies, 4.7%) discussed confounding on the effect modifier by another variable,^52–56^ while others (two studies, 1.8%) included multiple community characteristics as effect modifiers in the second stage of the Bayesian hierarchical model.^56,57^ Studies evaluated the health effects of multiple pollutants with varying methods (citations as examples and not exclusive): conducting separate analyses for each pollutant using single-pollutant models,^58^ conducting separate analyses for each pollutant while including other air pollutants as confounders (e.g., including PM_2.5_ total mass as a confounder when evaluating the effect of PM_2.5_ constituents), conducting one analysis by including all pollutants in the same multiple-pollutant models and interpreting the coefficients of all air pollutants from the same model,^59,60^ and employing mixture methods to explore the effect of changing all pollutant levels simultaneous (e.g., quantile g-computation).^61,62^

#### Mediation analysis studies

None of the nine studies conducting mediation analysis clearly stated the conceptual framework (i.e., decomposition vs. causal mediation framework) under which their analytical method was developed. However, Li et al^47^ stated that they invoked the weaker interpretation for the effect of RE introduced by VanderWeele and Robinson,^29^ which focused on the change in disparity after changing the mediator distributions across RE groups, and provided some context for the conceptual framework behind their study. Based on analytical method types defined in VanderWeele 2016,^63^ three studies utilized the regression-based difference method with the STATA gsem command or without using any software package,^48,49,51^ four utilized the regression-based product method with the “mediation” package in R or with STATA,^44–47^ and the other two used the Blinder-Oaxaca decomposition analysis with the Oaxaca package in R and Oaxaca command in STATA.^43,50,64^ Only four studies explicitly discussed the assumptions behind their mediation analysis, where Li et al,^47^ Jones et al,^44^ and Song et al^45^ mentioned that they assumed no reverse causation among exposure, outcome and mediator, nor unmeasured confounding. Benmarhnia et al^43^ mentioned that they assumed no exposure-induced confounder of the mediator-outcome relationship. Since Howe et al advocated the use of causal diagrams to study health disparities in their 2022 publication,^65^ two of the four subsequent papers have used Directed Acyclic Graphs to describe their study designs and assumptions.^47,50^ Although it is possible and important to consider exposure-mediator(s) interactions in such analyses and provide four-way decomposition of the effects,^66^ only Li et al^47^ considered potential interactions between RE and air pollution on the health outcome, but they only provided the proportion mediated without revealing details of their decompositions.

### Interpretation of results

Nearly all studies included a discussion of RE findings, except for one EM study, which observed no significant differences across RE. However, the use of conceptual frameworks to justify the inclusion of RE or to contextualize their discussions was less prevalent. Exposure disparity studies were more likely to employ conceptual frameworks (26 of 65 studies, 40.0%) compared with EM studies (27 of 107 studies, 25.2%). All nine studies that included exploration of air pollution as a potential mediator in the outcome disparity across RE provided subject matter rationales for such exploration and discussed the inferences of their results.

There were 18 exposure disparity studies and six EM studies that interpreted or reported coefficients of covariates other than the primary exposure of interest, contradicting the purpose of descriptive study and contributing to the “table-2 fallacy” problem, as elaborated later.^67^ We did not consider studies that interpreted coefficients of another air pollutant as suffering from the “table-2 fallacy.”

## Discussion

We provided an overview of the landscape of air pollution-related studies that utilized RE in California, evaluated the prevalence of some methodological challenges, and provided corresponding recommendations (Table 4).

**Table 4.** Methodological recommendations for air pollution studies considering race and ethnicity

Methodological considerations	Recommendations
Operationalization and conceptual framework of race and ethnicity (RE)	1. Provide theoretical justifications for the RE categorization and avoid categorizations that are too coarse 2. Provide conceptual frameworks on the role of RE in air pollution-related studies a. Avoid simply considering RE as a confounder b. Consider RE as a marker of exposure to structural racism c. Utilize metrics that capture structural racism in different domains
Air pollution estimation	1. Explore dynamic exposure considering activity space 2. Consider exact addresses combined with grid-based exposures instead of administrative units as the spatial unit of analysis 3. Explore spatial heterogeneity of air pollution-health association
Exposure disparity studies	1. Avoid adjusting/standardizing for other variables when conducting descriptive studies
Effect modification analysis studies	1. Distinguish between interaction and effect modification, especially its implications for confounding adjustment 2. Conduct a formal heterogeneity test when using stratified analyses 3. Avoid interpreting coefficients of covariates other than the exposure of interest (table-2 fallacy)
Mediation analysis studies	1. More utilization is encouraged 2. Clarify the hypothetical interventions of interest (or lack of thereof) and adopt relevant mediation or decomposition frameworks accordingly 3. Clearly state the model assumptions behind the analysis and consider exposure-mediator interactions when possible

### Operationalization and conceptual framework of RE

Our study revealed similar patterns and challenges in the operationalization of RE as those identified by Martinez et al.^41^ While Martinez et al found that most studies did not provide clear RE measurement details (up to 81%), we found that RE categorization schemes often collapsed RE into fewer categories without clear theoretical justifications (e.g., RE categories could serve as a general proxy for factors impacting health while the “other” group does not support such justification). This could be an attempt to achieve large enough sample sizes in each subgroup for statistical inference, but no real, interpretable inference can be made without a clear theoretical justification of creating such subgroups. Including coarse RE categories based on obscure definitions appeared in our findings through the prevalent use of broad ethno-racial constructs, and such coarse aggregation may mask important heterogeneity within groups. For example, Ard et al^68^ found meaningful differences in exposures to air pollution across Hispanic Americans subgroups such as Mexican, Dominican, Cuban, and Puerto Ricans, potentially due to historical spatial isolation and different employment activities of these subgroups. Moreover, the tendency to treat Hispanic ethnicity as a de facto racial category in many studies (as shown in combinations like “Asian, Black, Hispanic, White”) reflects the field’s ongoing challenge with properly distinguishing between racial and ethnic classifications. In the United States, although both RE are social constructs related to identity and ancestry, racial boundaries tend to rely more on phenotypic traits such as skin tone or other physical characteristics, whereas ethnic boundaries are more closely tied to language, religion, and shared beliefs,^41^ leading to different patterns of exposure and vulnerability towards air pollution between RE. In sum, providing theoretical justifications for the RE categorization and avoiding categories that are too coarse can be the first step toward the right direction (Table 4). In the case where RE categories need to be collapsed to avoid small sample sizes, collapsing RE categories should be theoretically driven.

There is also a lack of proper conceptualization of RE in air pollution-related studies. Only 33.1% of studies included a discussion of conceptual framework for the inclusion of RE, which is even lower in EM studies. While Casey et al^25^ found that 50% of general environmental justice research published between 2018 and 2021 explicitly utilized an EJ conceptual framework, our analysis of air pollution studies for that same period found an even lower prevalence of only 28.8%. Compared with the broader environmental health research field, air pollution research with RE needs to improve its integration of environmental justice theories into study design and interpretation.

Drawing from previous literature,^10,15^ a robust conceptualization of RE in air pollution research needs to consider several key points. First, we need to move beyond treating race as a simple demographic variable and instead examine it as a marker of exposure to structural racism, which shapes both environmental exposures and vulnerability to their health effects. Hicken et al.^10^ emphasized that race categories have different social meanings across locations and time periods, suggesting that researchers should carefully consider local sociopolitical contexts when interpreting racial patterns in air pollution exposures and health outcomes. Second, we want to underscore the importance of developing and incorporating measures of structural racism specific to environmental health. Rather than simply documenting racial disparities, three studies in our review utilized measures of structural racism, such as racial segregation, which links to discriminatory practices and policies that created and maintained these disparities, ultimately informing more targeted interventions. Although understanding health disparities across conventional individual-level and demographic covariates is important, it is equally crucial to integrate RE within the framework of contemporary structural racism theories. For California with its diverse population, these metrics must reflect the state’s unique sociopolitical landscape, such as metrics for immigrant enclaves, linguistic isolation, or state-specific redlining legacies, which may differ from the predominantly Black-white binary frameworks often used in other US regions. Developing these metrics is also relevant for other parts of the US Emerging literature in social epidemiology has demonstrated the use of metrics directly measuring structural racism to better explain observed racial differences across typologies of structural racism, such as housing.^69–72^

### Air pollution estimation-related methodological considerations

We have three methodological recommendations related to air pollution estimation methods (Table 4). First, we recommend exploring the consideration of dynamic exposures in assessing health disparities across RE related to air pollution, which incorporates individual activity patterns in exposure assessment instead of the static approach relying solely on the place of residence. This concept of activity space has emerged in the past two decades in social epidemiology and has been used in air pollution epidemiology recently.^73^ A recent study identified a higher effect of NO_2_ on insulin resistance among Hispanic participants only after considering a dynamic exposure,^74^ highlighting the importance of dynamic exposures in evaluating health disparities across RE. Second, we recommend exploring the amplitude of bias introduced by using administrative units in spatial analysis, as exact coordinates of study participants are becoming available in some cohorts. Different geographical resolutions were preferred for different types of study, with more grid-based estimates in exposure disparity studies and more administrative unit-level estimates in EM studies. As most health outcomes and population data were collected for varying administrative units, the observed pattern is not surprising. However, administrative boundaries were not the optimal polygons to use in spatial analysis given their irregular shapes and sizes, leading to the modifiable areal unit problem where results can change depending on the size and shape of the boundaries,^75^ as shown by an exposure disparity study identified in this review.^76^ Using exact addresses can mitigate this problem, but individual activity patterns further complicate exposure assessment, and recent studies advocate for dynamic modeling that incorporates these activity patterns.^74^ Third, we encourage the implementation of spatially varying coefficient models^77–79^ to identify areas in which air pollution-related health disparity across RE are more (or less) pronounced, instead of focusing exclusively on one effect estimate across all spatial units, as done in all studies included in our review.

### Methodological considerations specific to exposure disparity studies

We also want to discourage the adjustment for other variables in descriptive studies or exposure disparity studies in our context. In this review, 27.7% (18 of 65) exposure disparity studies interpreted coefficients of variables (e.g., SES) other than RE. The rationale for adjusting for other variables when conducting descriptive studies is not clear, and some scholars have argued that descriptive studies should not condition or standardize on other variables, as one goal of descriptive studies is to describe the world as it is.^80^ Standardizing on covariates such as age or gender, for example, creates a nonexistent pseudo-population in which RE subgroups have the same distribution of age and gender as the reference population, which can be seen as misleading. Such practice, sometimes referred to as “ritualistic adjustment” has been described^28^ in the social epidemiology literature and is still prevalent in the studies we identified in this review.

### Methodological considerations specific to effect modification analysis studies

In 107 studies evaluating RE as effect modifiers, the distinction between interaction and EM needs to be emphasized, especially their epidemiological interpretations. As defined by VanderWeele, an interaction “requires the effect of two exposures together to be different from the combination of the two effects considered separately,” while EM “is defined in terms of the effect of one intervention [exposure] varying across strata of a second variable [effect modifier].”^81^ An important distinction between interaction and EM is the role of confounding. For interactions, studies need to account for confounding between the exposure and outcome. For EM, studies should not account for confounding between the effect modifier and outcome because this confounding is part of the EM.^81^ Unfortunately, interaction is also a widely used statistical term, and studies tend to use it without considering their epidemiological interpretation. A total of 23 (21.5%) EM studies in our review only described their approach using terms like interaction or stratification, likely referring to the analytical methods. Explicitly describing their analysis as evaluating EM or effect heterogeneity will clarify the actual purpose of their analyses. On the other hand, 4 (3.7%) studies discussed residual confounding on their effect modifiers, which was unnecessary because confounding between the effect modifier and outcome should be considered as part of the EM. Besides, 2 (1.9%) studies^56,57^ included multiple effect modifiers in the second stage of the Bayesian hierarchical model, “adjusting for other effect modifiers when evaluating the EM,” which is not aligned with the definition of EM. As opposed to causal interaction, no manipulation is targeted for effect modifiers, and the concept of confounding does not apply here.

Another important aspect in reporting EM is conducting formal heterogeneity tests, such as Cochran’s Q test, Wald test, or conducting regression analysis using interaction terms, to evaluate whether the effects are truly different across RE strata.^82,83^ As pointed out by Gelman and Stern in 2006, a common error when comparing two or more effect estimates (i.e., across RE strata) is to compare their degrees of statistical significance rather than formally evaluating whether the estimates differ statistically.^84^ When a study failed to reject the homogeneous effect hypothesis, a single effect estimate for all subgroups combined should be reported.^82^ Since the heterogeneity test results can change across effect‑measure scales (e.g., additive vs. multiplicative) and the appropriate scale varies depending on the biological mechanism being evaluated, Knol and VanderWeele recommended reporting stratum-specific effect estimates and formal heterogeneity test results on both scales.^85^ In our review, 70 (65.4%) EM studies conducted a formal heterogeneity test, and 62 of them reported stratum-specific effect estimates, irrespective of whether they detected significant heterogeneity. This proportion is consistent over the three periods (2000–2010, 2011–2019, and 2020–2025). The prevalence of conducting formal heterogeneity tests is higher than the 34% identified in epidemiological studies of disparities in toxic chemical exposure and neurodevelopmental outcomes by Payne-Sturges et al,^86^ and 53% identified in 138 medical and epidemiological studies addressing interaction between 2001 and 2007 by Knol et al.^87^ We would like to encourage using formal heterogeneity tests, reporting stratum-specific results when heterogeneity is detected, and reporting findings from both additive and multiplicative scales.

In population-based research, presenting and interpreting coefficients of covariates other than the exposure of interest from a multivariable model, such as confounders, leads to a scenario called “table-2 fallacy.”^67^ Interpreting coefficients of covariates other than the exposure of interest is problematic because the set of confounders that need to be adjusted to obtain a causal relationship with the outcome is likely different for the exposure of interest and the covariate whose coefficient is being interpreted. This might not apply when multiple air pollutants were considered as confounders of each other and included in the same multivariate model (e.g., multiple-pollutant models) as many air pollutants share the same confounders for health outcomes; thus, we excluded such studies in the discussion of table-2 fallacy and discussed multiple pollutants in the next paragraph. The prevalence of table-2 fallacy is relatively low in EM analysis studies included in our review, with only 5.6% of studies (6 of 107) interpreted coefficients of nonair pollutant variable.^88–91^ Recommendations regarding how to avoid the table-2 fallacy have been described elsewhere.^26^

### Methodological considerations specific to mediation analysis studies

While we identified fewer studies that considered a mediation framework, such approaches can be particularly helpful at quantifying how much inequalities across RE subgroups are due to air pollution for a given health outcome. The use of mediation techniques to analyze the drivers of SES inequalities in health is increasing,^31^ supported by newly developed guidelines for reporting and considerations in analytical planning.^42,65^ These recommendations are to use causal graphs when developing the analytical plan, to report the conceptual framework and model assumptions, and to consider interactions between exposure and mediators, which were underutilized in studies identified in our review. Treating RE as the main exposure of interest has been criticized due to the violation of the consistency assumption and the lack of manipulability (VanderWeele 2018), so some scholars have proposed to shift the manipulability criteria toward the mediator, such as a given air pollutant. In this context, it would be more accurate to focus on a decomposition framework rather than a causal mediation framework. For example, estimating a natural indirect effect requires a hypothetical manipulation of the exposure, while a decomposition only requires a change in the distribution of the mediator(s). Recent decomposition techniques for such setting have been proposed,^92^ and we argue that it would be particularly interesting to implement such techniques to study health outcomes of multiple air pollutants.

### Limitations

There are some limitations in this review. First, we focused on the methodological choices and assumptions behind air pollution-related studies that utilized RE and did not provide summaries of observed disparity across racial and ethnic groups. However, previous reviews already provided detailed summaries of disparity on the associations of air pollution and pregnancy outcome,^93^ PM and birth outcomes,^9,27^ and ozone and mortality or hospital admission.^94^ Second, our focus on RE usage in air pollution studies focused on California may not reflect RE usage in air pollution nationally or in other US regions. California has a diverse population that differs from other US regions. For example, California has a relatively lower proportion of Black residents compared to major cities on the East Coast or the Southeastern United States, and so the prevalence of certain practices, such as the use of conceptual frameworks or structural racism metrics, may reflect dynamics specific to California’s demographic and political context. That said, California provides a particularly important lens for studying the dynamics of ethnicity, especially for Hispanic and immigrant populations. More broadly, racial categories in the United States carry specific historical and structural meaning tied to institutionalized racism that does not map directly onto racial or ethnic classifications in other national contexts. Caution is warranted when applying these findings outside the United States. Besides, many of the core methodological suggestions that we propose, including grounding social categories in explicit conceptual frameworks, distinguishing their roles as confounders, effect modifiers, or main exposures, and applying appropriate mediation and decomposition methods, are relevant to researchers studying other social determinants of health (e.g., socioeconomic position, gender, caste, and immigration status) regardless of the national contexts. Third, we also did not exhaust all methodological considerations in air pollution-related health studies that used RE, but this review should provide a good start for future discussions and directions. For example, we did not discuss the intersectionality framework such as the multilevel analysis of individual heterogeneity and discriminatory accuracy, which could be incorporated into considerations of RE in air pollution-related studies.^95^ It enables the assessment of inequalities across multidimensional social strata, including RE and factors such as socioeconomic position and rurality, which might affect the influence of RE on both differential exposure and vulnerability. In contrast, many existing studies rely on composite measures such as the Social Vulnerability Index, which integrate several dimensions of social vulnerability but can mask the distinct effects of RE. Finally, we also limited our review to six criteria air pollutants, AQI, and wildfire smoke to restrict the review to a manageable size and disregarded other air pollutants. However, the methodological challenges and recommendations discussed in this review should apply to other air pollutants.

## Conclusions

In this scoping review, we synthesized the evolving landscape of air pollution-related health studies in California that incorporated racial and ethnic considerations. Our analysis, including studies from 2000 to 2025, revealed a growing trend in research exploring air pollution disparities across RE groups and examining RE as effect modifiers in air pollution-outcome relationships. However, studies investigating the mediating role of air pollution in outcome disparities across RE remain underrepresented. Through our systematic approach, we identified key methodological challenges and provided targeted recommendations for researchers. This work serves a double purpose: guiding future research endeavors and equipping policymakers and the public with a nuanced understanding of the conceptual frameworks and assumptions underlying these studies. By addressing these critical aspects, we aim to enhance the quality and impact of air pollution-related health research, ultimately contributing to more equitable and effective public health strategies.

## Conflicts of interest statement

The authors declare that they have no conflicts of interest with regard to the content of this report.

## Supplementary Material
