# Supplementary material for: The use of race and ethnicity in air pollution epidemiology and methodological recommendations: A scoping review focusing on California
Source: Environ Epidemiol. 2026 Jun 1;10(3):e485. doi: 10.1097/EE9.0000000000000485 (PMC13232931; doi:10.1097/EE9.0000000000000485)
Supplement: Supplementary file 1 [file ee9-10-e485-s001.pdf]

Supplementary materials

Supplementary text 1. Identification of potentially eligible original articles cited in relevant reviews.

Briefly, we searched the four databases with similar search terms as described above but primed to identify reviews as compared to focusing on original articles (Table S3). This search yielded 207 potentially relevant review articles, and we reduced this to 48 through abstract and title review based on the eligibility/exclusion criteria (Table S1). We also identified four relevant California Air Resources Board (CARB) reports through abstract and title review. Finally, each researcher read half of the relevant reviews and identified potentially eligible original articles cited. Two researchers independently conducted the abstract and title screening. Conflicts were resolved through discussion. Prior to beginning the formal screening process, the two researchers underwent a calibration exercise using five review papers not included in the study sample to ensure consistency in their selection approach. Any discrepancies in paper selection during this exercise were discussed to resolve conflicts and establish a standardized screening criterion. Through this process, we identified 343 potentially relevant papers that we then added to title and abstract screening process described in Figure 2.

Supplementary text 2. Summary of studies excluded at the full-text review stage.

Among 109 excluded studies, reasons for exclusion included studies conducted exclusively outside of California (n=40), studies focused on air pollutants other than the six criteria air pollutants, wildfire, or air quality index (n=17), and studies that used RE as a confounder (n=20). Additional exclusions were made for non-peer-reviewed or non-human population studies (n=13), studies examining only a single racial and ethnic subpopulation (n=5), studies where air pollution was not considered as an exposure, mediator, or outcome (n=6), health impact assessments that solely used existing concentration-response functions (n=6), and simulation studies (n=2) (Figure 2).

Supplementary text 3. Items used to extract information in the survey

A. *Study Identification and Characteristics*
   1. **DOI:** Digital Object Identifier of the publication.
   2. **Title:** Full title of the published article.
   3. **First Author:** First and Last name of the first author.
   4. **Year of Publication:** The year the article was published.
   5. **Study Period:** Start Year
   6. **Study Period**: End Year
   7. **Geographic Area:** The location or region where the study was conducted.
   8. **Study Population:** Did the study consider a single race/ethnic group or more than one groups.
   9. **Sample Size:** The total number of participants in the study.
   10. **Study Type:** Exposure disparity, health disparity with effect modification, Health disparity with mediation analysis, or Other.

B. *Data Sources*
   11. **Primary Outcome:** The main outcome investigated.
   12. If the paper uses a health outcome, what is the unit of analysis for the outcome?
   13. What is the exposure?

C. *Air pollution related variables*
   14. **How was air pollution used in this study** (e.g. Exposure, Effect Modifier, mediator)?
   15. **Air Pollution Variable Used in the study** (e.g., $PM_{2.5}$, CO, O_3).
   16. **Source of Air Pollution Data** (monitoring data, model based, combination of monitoring data and model, other).
   17. **Geographic Unit of Air Pollution** (e.g., census tract, ZIP code).
   18. **Temporal Resolution of Air Pollution** (e.g., daily, monthly, annual average).

D. *Race/Ethnicity (R/E) Variable*
   19. **How was Race/Ethnicity used in the study** (Exposure, Effect modifier, Mediator).
   20. **Source of Race/Ethnicity Data** (Specific Data Source)**.**
   21. **How was R/E data collected?** (e.g., self-report).
   22. **Level of R/E Data** (e.g., individual level, proportion of R/E within an area).
   23. If index is used to capture R/E, name of the index
   24. If not an index, the specific categories used for the R/E variable.
   25. Did the authors combine any of the original race/ethnicity categories for their analysis?
   26. If yes, describe which groups were collapsed.
   27. Did the authors include race/ethnicity in any other form in the analysis (e.g., community-level R/E proportion as a confounder/region-level covariate)?
   28. If yes, what was the variable and its aggregation level?
   29. If yes, what was the source of this additional R/E variable?
   30. If yes, how was this additional R/E variable used in the analysis?
   31. Was the inclusion of this additional R/E variable justified?

32. If yes, what was the provided reason?

E. *Analytical Approach and Findings*

Exposure Disparity studies

33. If the study focused on exposure disparity, what was the primary analytical approach?
34. If descriptive metrics of air pollution were used, specify which ones (e.g., population-weighted average, coefficient of divergence).

Health Disparity study with Mediation

35. If it was a health disparity study with mediation analysis, what was the study design?

Health Disparity study with Effect Modification

36. If it was a health disparity study evaluating effect modification, what was the study design?
37. Did the authors explicitly use the term effect modificaiton/effect heterogeniety in the study?
38. Did any of the following occur in this study?
    - Including multiple community characteristics in a second stage model (e.g. time series model, bayesian heierarchical model)
    - Discussion of confounding on the effect modifier by other variable
39. If it is health disparity study with effect modification evaluation, what is the primary analytical approach?
40. If it is a health disparity study with effect modification evaluation, was a heterogeneity test conducted for R/E?
41. If a heterogeneity test was conducted, what test? (e.g. ANOVA, WALD test).
42. If they conducted a heterogeneous test, did they find a heterogeneous effect across R/E and reported stratum-specific effects?
43. Do the authors interpret coefficients of any other variable other than the exposure of interest (i.e., confounders) from the same model?

F. *Discussion and Interpretation*

44. Summarize Empirical Findings
45. Did the authors discuss their findings related to race/ethnicity?
46. Did they use a conceptual framework to explain their findings on R/E?
47. Summarize the discussion points from the paper around R/E (Tip: structural racism, environmental injustice)

Figure S1. Flowchart detailing the process at the abstract-screening and full-text review stage.

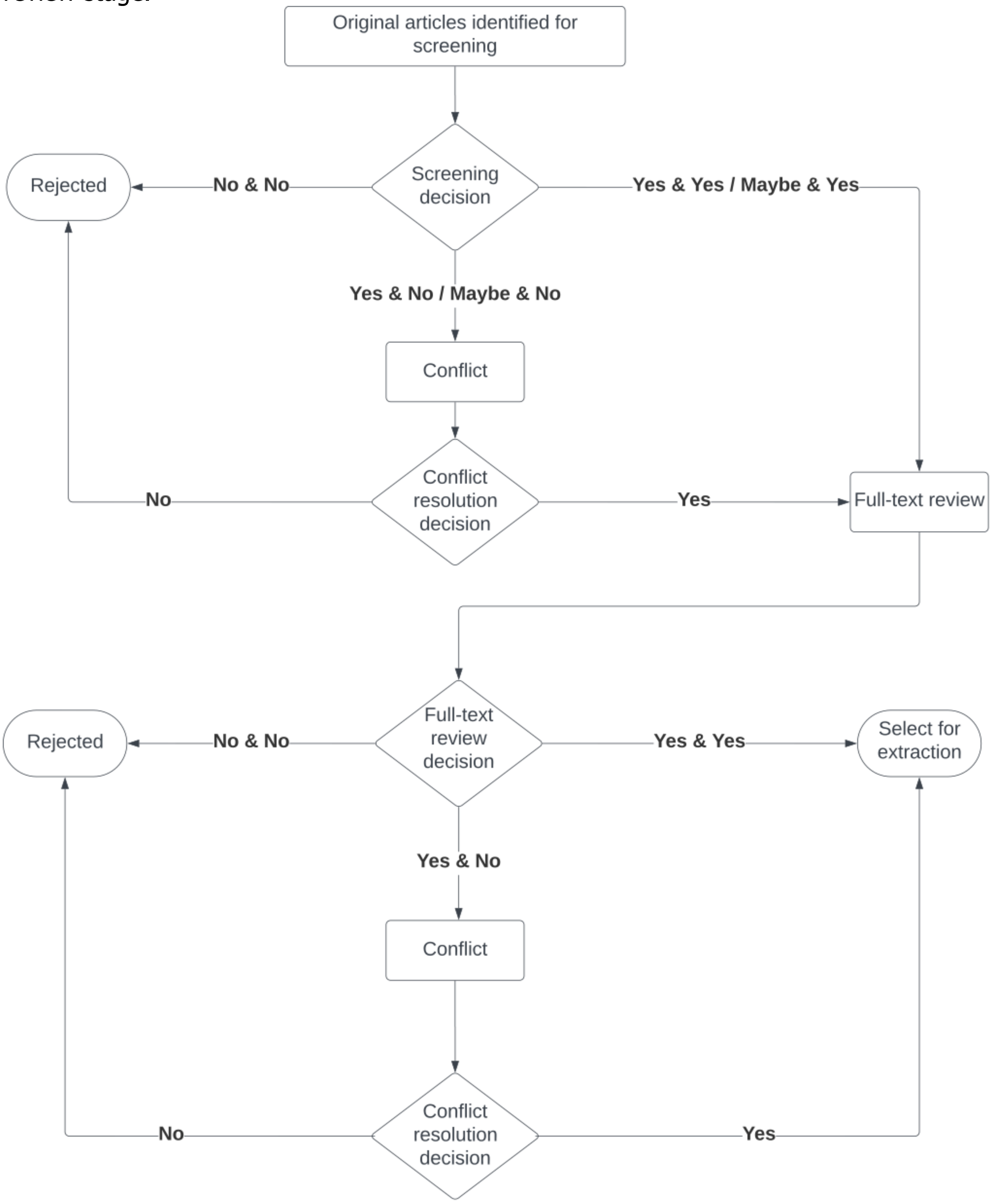

Table S1. Eligibility criteria and corresponding exclusion criteria for the scoping review.

Eligibility Criteria	Exclusion Criteria
1. English language	1. Not in English
2. Peer-reviewed empirical population studies published as journal articles	2. Reviews, commentaries, letters, editorials, news and case reports, conference papers, pre-prints
3. Publication year between 2000/1/1 and 2025/7/31	3. Published before 2000/1/1 or after 2025/7/31
4. Must be conducted within California or a general focus on the US	4. Exclusively focusing on an area outside of California (e.g., Europe, east coast of US or Florida)
5. Must use ambient air pollution (six criteria air pollutant, wildfire, air quality index) as exposure, outcome or mediator	5. Occupational cohorts, animal studies, health impact assessment, simulation studies, or non-population-based studies (i.e. those that involved experimental or randomized exposure schemes)
6. Must include race and ethnicity as exposure (when air pollution is the outcome) or effect modifier or mediator	6. Race and ethnicity are used as a confounder
	7. Air pollution is used as a confounder
	8. Focused on indoor air pollution, smoking, or ambient air pollutants other than ones specified.

Table S2. Search terms used for different databases for original articles.

Database	Search Terms
PubMed	("atmospheric pollutant"[tiab] OR "atmospheric pollutants"[tiab] OR "atmospheric pollution"[tiab] OR "air pollution"[tiab] OR "air pollutant"[tiab] OR "air pollutants"[tiab] OR "air quality"[tiab] OR "wildfire"[tiab] OR "particulate matter"[tiab] OR "PM"[tiab] OR "PM2.5"[tiab] OR "PM10"[tiab] OR "ozone"[tiab] OR "O3"[tiab] OR "carbon monoxide"[tiab] OR ("Lead"[tiab] AND "air"[tiab]) OR "sulfur dioxide"[tiab] OR "SO2"[tiab] OR "nitrogen dioxide"[tiab] OR "NO2"[tiab]) AND ("race"[tiab] OR "ethnicity"[tiab] OR "racial"[tiab] OR "ethnic"[tiab] OR "races"[tiab] OR "ethnicities"[tiab] OR "minority group"[tiab] OR "minority groups"[tiab]) AND ("US" OR "United States" OR "California" OR "USA" OR "America" OR "American") AND (English[Language]) NOT (Letter[Publication Type]) NOT (Comment[Publication Type]) NOT (Editorial[Publication Type]) NOT (Review[Publication Type]) NOT (News[Publication Type]) NOT (Case Reports[Publication Type])
EMBASE	('atmospheric pollut*':ab,ti OR 'air pollut*':ab,ti OR 'air quality':ab,ti OR 'wildfire':ab,ti OR 'particulate matter':ab,ti OR 'pm':ab,ti OR 'pm2.5':ab,ti OR 'pm10':ab,ti OR 'ozone':ab,ti OR 'o3':ab,ti OR 'carbon monoxide':ab,ti OR ('lead':ab,ti AND 'air':ab,ti) OR 'sulfur dioxide':ab,ti OR 'so2':ab,ti OR 'nitrogen dioxide':ab,ti OR 'no2':ab,ti) AND ("race$":ab,ti OR 'ethnicit*':ab,ti OR 'racial':ab,ti OR 'ethinic':ab,ti OR "minority group$":ab,ti) AND ('us' OR 'united states'/exp OR 'united states' OR 'california'/exp OR 'california' OR 'usa'/exp OR 'usa' OR 'america'/exp OR 'america' OR 'american'/exp OR 'american') AND ([article]/lim OR [article in press]/lim) AND [english]/lim AND [01-01-2000]/sd NOT [08-01-2025]/sd
Web of Science	((TI = ("atmospheric pollutant" OR "atmospheric pollutants" OR "atmospheric pollution" OR "air pollution" OR "air pollutant" OR "air pollutants" OR "air quality" OR "wildfire" OR "particulate matter" OR "PM" OR "PM2.5" OR "PM10" OR "ozone" OR "O3" OR "carbon monoxide" OR ("Lead" AND "air") OR "sulfur dioxide" OR "SO2" OR "nitrogen dioxide" OR "NO2")) OR (AB = ("atmospheric pollutant" OR "atmospheric pollutants" OR "atmospheric pollution" OR "air pollution" OR "air pollutant" OR "air pollutants" OR "air quality" OR "wildfire" OR "particulate matter" OR "PM" OR "PM2.5" OR "PM10" OR "ozone" OR "O3" OR "carbon monoxide" OR ("Lead" AND "air") OR "sulfur dioxide" OR "SO2" OR "nitrogen dioxide" OR "NO2"))) AND ((TI = ("race" OR "racial" OR "ethnicity" OR "ethnic" OR "races" OR "ethnicities" OR "minority group" OR "minority groups")) OR (AB = ("race" OR "racial" OR "ethnicity" OR "ethnic" OR "races" OR "ethnicities" OR "minority group" OR "minority groups"))) AND (CU = ("US" OR "United States" OR "California" OR "USA" OR "America" OR "American")) AND (LA = English)

	NOT DT=(Letter OR Comment OR Editorial Material OR Review OR News Item OR Case Reports)
CINHAL	(TI (("atmospheric pollutant" OR "atmospheric pollutants" OR "atmospheric pollution" OR "air pollution" OR "air pollutant" OR "air pollutants" OR "air quality" OR "wildfire" OR "particulate matter" OR "PM" OR "PM2.5" OR "PM10" OR "ozone" OR "O3" OR "carbon monoxide" OR "lead" AND "air" OR "sulfur dioxide" OR "SO2" OR "nitrogen dioxide" OR "NO2") AND ("race" OR "racial" OR "ethnicity" OR "ethnic" OR "races" OR "ethnicities" OR "minority group" OR "minority groups") AND ("united states" OR "california" OR "usa" OR "america" OR "american")) OR AB (("atmospheric pollutant" OR "atmospheric pollutants" OR "atmospheric pollution" OR "air pollution" OR "air pollutant" OR "air pollutants" OR "air quality" OR "wildfire" OR "particulate matter" OR "PM" OR "PM2.5" OR "PM10" OR "ozone" OR "O3" OR "carbon monoxide" OR "lead" AND "air" OR "sulfur dioxide" OR "SO2" OR "nitrogen dioxide" OR "NO2") AND ("race" OR "racial" OR "ethnicity" OR "ethnic" OR "races" OR "ethnicities" OR "minority group" OR "minority groups") AND ("united states" OR "california" OR "usa" OR "america" OR "american"))) NOT PT (Letter OR Comment OR Editorial OR Review OR news OR case reports)

Table S3. Search terms used for different databases for review articles that were used to identify relevant original articles to supplement the main search.

Database	Search Terms
PubMed	("atmospheric pollutant"[tiab] OR "atmospheric pollutants"[tiab] OR "atmospheric pollution"[tiab] OR "air pollution"[tiab] OR "air pollutant"[tiab] OR "air pollutants"[tiab] OR "air quality"[tiab] OR "wildfire"[tiab] OR "particulate matter"[tiab] OR "PM"[tiab] OR "PM2.5"[tiab] OR "PM10"[tiab] OR "ozone"[tiab] OR "O3"[tiab] OR "carbon monoxide"[tiab] OR ("Lead"[tiab] AND "air"[tiab]) OR "sulfur dioxide"[tiab] OR "SO2"[tiab] OR "nitrogen dioxide"[tiab] OR "NO2"[tiab]) AND ("race"[tiab] OR "ethnicity"[tiab] OR "racial"[tiab] OR "ethnic"[tiab] OR "races"[tiab] OR "ethnicities"[tiab] OR "minority group"[tiab] OR "minority groups"[tiab]) AND ("US" OR "United States" OR "California" OR "USA" OR "America" OR "American") AND (English[Language]) AND (Review[Publication Type]) NOT (Letter[Publication Type]) NOT (Comment[Publication Type]) NOT (Editorial[Publication Type]) NOT (News[Publication Type]) NOT (Case Reports[Publication Type])
EMBASE	('atmospheric pollut*':ab,ti OR 'air pollut*':ab,ti OR 'air quality':ab,ti OR 'wildfire':ab,ti OR 'particulate matter':ab,ti OR 'pm':ab,ti OR 'pm2.5':ab,ti OR 'pm10':ab,ti OR 'ozone':ab,ti OR 'o3':ab,ti OR 'carbon monoxide':ab,ti OR ('lead':ab,ti AND 'air':ab,ti) OR 'sulfur dioxide':ab,ti OR 'so2':ab,ti OR 'nitrogen dioxide':ab,ti OR 'no2':ab,ti) AND ("race$":ab,ti OR 'ethnicit*':ab,ti OR 'racial':ab,ti OR 'ethinic':ab,ti OR "minority group$":ab,ti) AND ('us' OR 'united states'/exp OR 'united states' OR 'california'/exp OR 'california' OR 'usa'/exp OR 'usa' OR 'america'/exp OR 'america' OR 'american'/exp OR 'american') AND [english]/lim AND [01-01-2000]/sd NOT [08-01-2025]/sd AND ([letter]/lim OR [review]/lim)
Web of Science	((TI = ("atmospheric pollutant" OR "atmospheric pollutants" OR "atmospheric pollution" OR "air pollution" OR "air pollutant" OR "air pollutants" OR "air quality" OR "wildfire" OR "particulate matter" OR "PM" OR "PM2.5" OR "PM10" OR "ozone" OR "O3" OR "carbon monoxide" OR ("Lead" AND "air") OR "sulfur dioxide" OR "SO2" OR "nitrogen dioxide" OR "NO2")) OR (AB = ("atmospheric pollutant" OR "atmospheric pollutants" OR "atmospheric pollution" OR "air pollution" OR "air pollutant" OR "air pollutants" OR "air quality" OR "wildfire" OR "particulate matter" OR "PM" OR "PM2.5" OR "PM10" OR "ozone" OR "O3" OR "carbon monoxide" OR ("Lead" AND "air") OR "sulfur dioxide" OR "SO2" OR "nitrogen dioxide" OR "NO2"))) AND ((TI = ("race" OR "racial" OR "ethnicity" OR "ethnic" OR "races" OR "ethnicities" OR "minority group" OR "minority groups")) OR (AB = ("race" OR "racial" OR "ethnicity" OR "ethnic" OR "races" OR "ethnicities" OR "minority group" OR "minority groups"))) AND (CU = ("US" OR "United States" OR "California" OR "USA" OR "America" OR "American")) AND (LA = English)

	AND DT = (Review) NOT DT=(Letter OR Comment OR Editorial Material OR News Item OR Case Reports)
CINHAL	(TI (("atmospheric pollutant" OR "atmospheric pollutants" OR "atmospheric pollution" OR "air pollution" OR "air pollutant" OR "air pollutants" OR "air quality" OR "wildfire" OR "particulate matter" OR "PM" OR "PM2.5" OR "PM10" OR "ozone" OR "O3" OR "carbon monoxide" OR "lead" AND "air" OR "sulfur dioxide" OR "SO2" OR "nitrogen dioxide" OR "NO2") AND ("race" OR "racial" OR "ethnicity" OR "ethnic" OR "races" OR "ethnicities" OR "minority group" OR "minority groups") AND ("united states" OR "california" OR "usa" OR "america" OR "american")) OR AB (("atmospheric pollutant" OR "atmospheric pollutants" OR "atmospheric pollution" OR "air pollution" OR "air pollutant" OR "air pollutants" OR "air quality" OR "wildfire" OR "particulate matter" OR "PM" OR "PM2.5" OR "PM10" OR "ozone" OR "O3" OR "carbon monoxide" OR "lead" AND "air" OR "sulfur dioxide" OR "SO2" OR "nitrogen dioxide" OR "NO2") AND ("race" OR "racial" OR "ethnicity" OR "ethnic" OR "races" OR "ethnicities" OR "minority group" OR "minority groups") AND ("united states" OR "california" OR "usa" OR "america" OR "american")))

Table S4. Combinations of RE categories considered in studies.

RE Categories[1]	n (%)
Asian, Black, Hispanic, White	10 (5.8%)
Hispanic, NH Black, NH White	10 (5.8%)
Black, Other, White	7 (4%)
Hispanic, NH Asian, NH Black, NH White	7 (4%)
Black, White	6 (3.5%)
Hispanic, NH Black, NH White, Other	6 (3.5%)
Black, non-Black	5 (2.9%)
NH Black, NH White	5 (2.9%)
Black, Hispanic, White	4 (2.3%)
Chinese American, Hispanic, NH Black, NH White	4 (2.3%)
Hispanic, NH Asian/PI, NH Black, NH White	4 (2.3%)
Asian, Black, Hispanic, NH White, Other	3 (1.7%)
Asian, Black, Hispanic, Native American, White	3 (1.7%)
Black, Hispanic, Japanese American, Native Hawaiian, White	3 (1.7%)
Black, Hispanic, Japanese American, White	3 (1.7%)
Black, NH White	3 (1.7%)
Hispanic, NH Asian, NH Black, NH White, Other	3 (1.7%)
Hispanic, NH White	3 (1.7%)
American Indian, Asian, Black, White	2 (1.2%)
Asian, Hispanic, NH Black, NH White	2 (1.2%)
Black, Chinese American, Hispanic, White	2 (1.2%)
Hispanic AIAN, Hispanic Asian, Hispanic Black, Hispanic Multiple, Hispanic NHPI, Hispanic Other, Hispanic White, NH AIAN, NH Asian, NH Black, NH Multiple, NH NHPI, NH Other, NH White	2 (1.2%)
Hispanic, NH AIAN, NH Asian, NH Black, NH NHPI, NH Other	2 (1.2%)
Hispanic, NH Black	2 (1.2%)
Hispanic, NH Black, NH Other, NH White	2 (1.2%)
Hispanic, non-Hispanic	2 (1.2%)
non-White	2 (1.2%)
non-White, White	2 (1.2%)
AIAN, Asian, Black, Hispanic, Multiple, NH White, NHPI, Other	1 (0.6%)
AIAN, Asian, Hispanic, NH Black, NH White, NHPI, Other, Unknown	1 (0.6%)
AIAN, Asian/PI, Black, Hispanic, White	1 (0.6%)
AIAN, Black, Chinese American, Hawaiian, Japanese American, Other, White	1 (0.6%)
AIAN, Black, Hispanic, Other	1 (0.6%)
AINA, Asian, Black, Hispanic, Multiple, Other, PI, White	1 (0.6%)
AINA, Asian, Black, Hispanic, NH White	1 (0.6%)

AINA, Asian, Black, Hispanic, NH White, NHPI	1 (0.6%)
AINA, Asian, Black, Hispanic, Other, PI, White	1 (0.6%)
AINA, Asian/PI, Black, Hispanic, NH, White	1 (0.6%)
American Indian, Asian, Black, Hispanic, NH White, Other, PI	1 (0.6%)
American Indian, Asian, Black, Hispanic, White	1 (0.6%)
American Indian, Hispanic, NH Asian/PI, NH Black, NH White	1 (0.6%)
Asian, Black, Hispanic White, NH White, Other	1 (0.6%)
Asian, Black, Hispanic, Multiple, Native American/PI, NH White	1 (0.6%)
Asian, Black, Hispanic, NH, NHPI, Other, White	1 (0.6%)
Asian, Black, Hispanic, Native American, NH White, Other	1 (0.6%)
Asian, Black, Hispanic, Native American, Other, White	1 (0.6%)
Asian, Black, Hispanic, Native American, PI, White	1 (0.6%)
Asian, Black, Hispanic, Other	1 (0.6%)
Asian, Black, Hispanic, Other, White	1 (0.6%)
Asian, Black, Hispanic, non-White	1 (0.6%)
Asian, Black, Multiple, Other, White	1 (0.6%)
Asian, Hispanic, Multiple, NHPI, PI, White	1 (0.6%)
Asian, Hispanic, NH (AIAN + Asian + PI), NH Black, NH White	1 (0.6%)
Asian/PI, Black, Hispanic, NH White, Other	1 (0.6%)
Asian/PI, Black, Hispanic, Native American, Other, White	1 (0.6%)
Asian/PI, Black, Hispanic, Other, White	1 (0.6%)
Asian/PI, Hispanic, NH Black, NH White	1 (0.6%)
Asian/PI, Hispanic, NH Black, NH White, Other	1 (0.6%)
Black, Cuban, Dominican, Hispanic, Hispanic Black, Hispanic White, Mexican, Puerto Rican, White	1 (0.6%)
Black, Hispanic	1 (0.6%)
Black, Hispanic, NH Asian, NH White, Other	1 (0.6%)
Black, Hispanic, Other	1 (0.6%)
Black, Hispanic, Other, White	1 (0.6%)
Black, Hispanic, non-White, White	1 (0.6%)
Black, Native American	1 (0.6%)
Black, Other	1 (0.6%)
Everyone other than NH White	1 (0.6%)
Hispanic, Mexican-American, NH Black, NH White, Other	1 (0.6%)
Hispanic, NH AIAN, NH Asian, NH Black, NH NHPI, NH White	1 (0.6%)
Hispanic, NH AIAN, NH Asian, NH Black, NH White	1 (0.6%)
Hispanic, NH AIANn, NH Asian/PI, NH Black, NH White, Unknown	1 (0.6%)
Hispanic, NH American Indian, NH Asian/PI, NH Black, NH White, Other	1 (0.6%)
Hispanic, NH Asian, NH Black	1 (0.6%)

Hispanic, NH Asian, NH Black, NH Native American or Aleut, NH Other, NH White	1 (0.6%)
Hispanic, NH Asian, NH Black, NH Native American, NH Other, NH PI, POC	1 (0.6%)
Hispanic, NH Asian, NH Black, NH Native American, NH White	1 (0.6%)
Hispanic, NH Asian, NH Black, NH Native American, NH White, Other	1 (0.6%)
Hispanic, NH Asian, NH Black, NH Other, NH White	1 (0.6%)
Hispanic, NH Asian, NH Black, NH White, POC	1 (0.6%)
Hispanic, NH Asian, NH Black, NH White, non-White	1 (0.6%)
Hispanic, NH Asian, NH White, NHBlack	1 (0.6%)
Hispanic, NH Asian/PI, NH Black, NH Other, NH White	1 (0.6%)
Hispanic, NH Asian/PI, NH Black, NH White, Other	1 (0.6%)
Hispanic, NH Black, NH East Asian, NH Other, NH South Asian, NH White	1 (0.6%)
Hispanic, NH Black, NH Minority	1 (0.6%)
Hispanic, White	1 (0.6%)
Minority RE groups, NH White	1 (0.6%)
Minority RE groups, Non-Minority	1 (0.6%)
NH Black	1 (0.6%)
NH White, non-White	1 (0.6%)
Other, White	1 (0.6%)
POC, White	1 (0.6%)
White	1 (0.6%)

[1] Abbreviations used:
AIAN: American Indian or Alaskan Native
ASIAN/PI: Asian or Pacific Islander
NHPI: Native Hawaiian or Pacific Islander
NH: non-Hispanic
POC: People of Color

Table S5. Number of air pollutants considered across study types.

Study Characteristics	All Studies (N = 178*)	Exposure Disparity Studies (N = 65)	EM Analysis Studies (N = 107)	Mediation Analysis Studies (N = 9)
Single Pollutant	97 (54.5%)	39 (60.0%)	56 (52.3%)	4 (44.4%)
Two Pollutants	38 (21.3%)	18 (27.7%)	18 (16.8%)	2 (22.2%)
Three Pollutants	21 (11.8%)	4 (6.2%)	16 (15.0%)	2 (22.2%)
Four or more pollutants	22 (12.4%)	4 (6.2%)	17 (15.9%)	1 (11.1%)

*Three studies were classified into two study types simultaneously, leading to differences in total number of studies and sum of studies across study types in some rows.

Table S6. Air pollutants considered across study type

Air Pollutant#	All Studies (N=178*)	Exposure Disparity Studies (N=65)	EM Analysis Studies (N=107)	Mediation Analysis Studies (N=9)
**Particulate matter (PM)**	135 (75.8%)	43 (66.2%)	86 (80.4%)	8 (88.9%)
$PM_{2.5}$	130 (73%)	41 (63.1%)	83 (77.6%)	8 (88.9%)
PM_{10}	21 (11.8%)	7 (10.8%)	14 (13.1%)	0 (0.0%)
$PM_{10-2.5}$	2 (1.1%)	0 (0.0%)	2 (1.9%)	0 (0.0%)
Ultrafine Particles	6 (3.4%)	2 (3.1%)	4 (3.7%)	0 (0.0%)
**NO_2**	61 (34.3%)	25 (38.5%)	34 (31.8%)	3 (33.3%)
**Ozone**	38 (21.3%)	8 (12.3%)	28 (26.2%)	3 (33.3%)
**CO**	15 (8.4%)	3 (4.6%)	11 (10.3%)	1 (11.1%)
**SO_2**	9 (5.1%)	4 (6.2%)	4 (3.7%)	1 (11.1%)
**Lead**	5 (2.8%)	2 (3.1%)	3 (2.8%)	0 (0.0%)
**$PM_{2.5}$ constituents or components**	16 (9%)	3 (4.6%)	13 (12.1%)	0 (0%)
**Source-specific air pollutants**				
Wildfire	16 (9%)	7 (10.8%)	9 (8.4%)	0 (0%)
Other source-specific air pollutants	11 (6.2%)	4 (6.2%)	7 (6.5%)	0 (0%)
**Air Quality Index**	2 (1.1%)	1 (1.5%)	1 (0.9%)	1 (11.1%)
**Other**	19 (10.7%)	4 (6.2%)	12 (11.2%)	3 (33.3%)

*Three studies were classified into two study types simultaneously, leading to differences in total number of studies and sum of studies across study types in some rows.

[#]Many studies considered multiple air pollutants simultaneously, leading to >100% sum across rows within each column. For example, the number of air pollutants explored in 65 exposure disparity studies was 104 because many studies considered multiple air pollutants simultaneously.
